# Resistance of Colorectal Cancer Stem Cells to Modern Therapies: A Systematic Review

**DOI:** 10.3390/ijms27146285

**Published:** 2026-07-15

**Authors:** Sarzhan Rustemov, Alina Kuandyk, Arailym Bertleuova, Syed Hani Abidi, Denis S. Bulanin

**Affiliations:** 1 Department of Medicine, Nazarbayev University School of Medicine, Kerey-Zhanibek Khandar 5/1, Astana 010000, Kazakhstan; sarzhan.rustemov@nu.edu.kz; 2 Department of Biomedical Sciences, Nazarbayev University School of Medicine, Kerey-Zhanibek Khandar 5/1, Astana 010000, Kazakhstan; alina.sabitova@nu.edu.kz (A.K.); arailym.bertleuova@nu.edu.kz (A.B.); hani.abidi@nu.edu.kz (S.H.A.)

**Keywords:** colorectal cancer, colorectal cancer stem cells (CRC-SCs), cancer stem cells (CSCs), therapeutic resistance, drug resistance, signaling plasticity, tumor microenvironment (TME), DNA damage response (DDR), epigenetic reprogramming

## Abstract

Colorectal cancer stem cells (CRC-SCs) contribute to treatment resistance, tumor recurrence and disease progression. Despite therapeutic advances, CRC-SCs frequently evade eradication and sustain tumor propagation. Although multiple molecular pathways have been implicated in this resistance, current preclinical evidence remains fragmented. This systematic review aims to synthesize preclinical evidence on the molecular mechanisms underlying CRC-SC resistance to modern anticancer therapies. A systematic search of PubMed, Scopus, Web of Science, and Cochrane Central Register of Controlled Trials was conducted to identify peer-reviewed original studies published between 2015 and 2025. Eligible studies investigated molecular mechanisms of CRC-SC resistance to chemotherapy, targeted therapy, immunotherapy, and other therapeutic modalities. Risk of bias was assessed using QUIN for in vitro studies and SYRCLE for in vivo studies. A total of 26 studies met the inclusion criteria. Synthesis of findings showed that CRC-SC resistance is driven by interconnected mechanisms, including adaptive signaling pathways, epigenetic reprogramming, enhanced DNA damage response, and protective interactions within the tumor microenvironment. Several studies reported that combination treatments targeting these mechanisms attenuated stemness characteristics and restored therapeutic sensitivity. Overall, CRC-SC resistance arises from multiple intrinsic and extrinsic mechanisms, supporting further preclinical and translational evaluation of combination strategies.

## 1. Introduction

Colorectal cancer (CRC) remains a major public health burden and a leading cause of cancer-related mortality [1]. In 2022, CRC accounted for 9.2% of cancer deaths among males and 9.4% among females worldwide [1]. Epidemiological projections suggest that, if current incidence trends persist, the global number of CRC cases will increase by approximately 22% by 2050 [2].

Despite substantial advances in early detection and multimodal treatment approaches, including surgery, radiotherapy, chemotherapy, and targeted therapies, CRC continues to be associated with high morbidity and mortality. Notably, approximately 30 to 40% of patients experience relapse following standard treatment, underscoring therapeutic resistance [3].

Accumulating evidence increasingly implicates cancer stem cells (CSCs) as central drivers of treatment failure, tumor recurrence, and disease progression [4,5]. CSCs constitute a distinct subpopulation of tumor cells characterized by self-renewal capacity, multipotent differentiation potential, tumor-initiating properties, and the ability to enter quiescent states [6]. These features enable CSCs to survive conventional therapies and repopulate the tumor, thereby contributing to relapse and metastatic dissemination [6]. Although CSCs remain incompletely characterized, they are increasingly recognized as key regulators across multiple stages of tumor development and therapeutic resistance [7].

The resistance of colorectal cancer stem cells (CRC-SCs) is recognized as a multifactorial process involving different mechanisms. These include signaling plasticity; enhanced DNA damage repair capacity and resistance to apoptosis, which promote cellular survival under genotoxic stress; epigenetic reprogramming, which maintains stemness; and dynamic interactions with the tumor microenvironment (TME), where hypoxic conditions, cytokine signaling, and stromal cell interactions collectively support CSC survival and protection [3,8,9,10,11,12]. However, these mechanisms are often described in isolation, and the exact molecular mechanisms underlying CRC-SC resilience remain only partially understood; in particular, there is a lack of a comprehensive synthesis integrating key processes, such as signaling plasticity, epigenetic changes, DNA repair, and microenvironmental crosstalk in CRC-SCs. Importantly, this review focuses exclusively on preclinical evidence, including in vitro and in vivo studies. Therefore, its findings should be interpreted as mechanistic and hypothesis-generating rather than as direct clinical recommendations. The current systematic review aims to address this gap by synthesizing evidence on the molecular mechanisms underlying CRC-SC resistance to modern anticancer therapies.

## 2. Materials and Methods

This study was conducted in accordance with the Preferred Reporting Items for Systematic Reviews and Meta-Analyses (PRISMA) guidelines [13] (Supplementary File S3; Supplementary File S4). The protocol for this review was registered in PROSPERO (CRD420251127132).

### 2.1. Search Strategy

Four electronic databases were searched (PubMed, Scopus, Web of Science, and Cochrane Central Register of Controlled Trials), using key terms such as “colorectal cancer,” “cancer stem cell,” “tumor-initiating cell,” “drug resistance,” and “therapy resistance,” covering the period from 1 January 2015, to 30 August 2025. The full line-by-line search strategies that were used in each of the four databases are provided in Supplementary File S1 and Supplementary File S2. Publication bias was reduced by searching unpublished literature using Google Scholar and OpenGrey. Additionally, backward and forward citation tracking was applied to included studies/articles that may have been missed during the database search.

### 2.2. Selection Criteria

Study eligibility was determined according to the following criteria: (1) studies specifically focused on colorectal cancer; (2) peer-reviewed original research investigating mechanisms of CSC resistance to modern therapies (drug therapies, immunotherapy, targeted therapy, etc.); and (3) publications dated between 2015 and 2025, a period corresponding to the clinical emergence and regulatory approval of chimeric antigen receptor T-cell (CAR-T) therapy by the U.S. Food and Drug Administration (FDA) in 2017 [14]. Studies were excluded based on the following criteria: (1) non-research-based publications, including conference abstracts, book chapters, commentaries, reviews, or editorials; (2) absence of accessible abstracts; (3) unavailability of full-text articles; and (4) publications in languages other than English.

### 2.3. Review Strategy

Titles and abstracts of identified records were imported into the Zotero reference management tool and screened by the first reviewer (S.R.) to exclude irrelevant studies and duplicates. A random subsample of 20% of titles and abstracts was independently screened by a second reviewer (A.B.) to ensure the accuracy of selection. Full-text articles were re-inspected (S.R. and A.B.) for relevance against the inclusion criteria. Discrepancies were resolved by involving a third reviewer (A.K.). The level of agreement between S.R. and A.B. was 77.8% (0.7778) and Cohen’s κ was 0.556. No automation tools, machine learning classifiers, or citation screening software were used at any screening stage.

### 2.4. Data Extraction

Data from included studies were extracted into a spreadsheet by the first reviewer (S.R.), including information on funding source, aim of the study, experimental models, duration of the experiment, outcome of interest, outcome measurement, control group presence, intervention, results before and after the intervention, inclusion/exclusion criteria, molecular mechanism affected, and key findings (Supplementary Table S5). The second reviewer (A.B.) ensured the accuracy at this stage by reviewing a random subsample of 20% of the extracted data. Discrepancies between reviewers were resolved through discussion involving a third reviewer.

### 2.5. Risk of Bias Assessment

Studies in this review comprised both in vitro and in vivo experimental components. Accordingly, risk of bias was assessed separately for each methodological domain to ensure an appropriate evaluation of study quality. For studies involving in vitro components, the Quality Assessment Tool for In Vitro Studies (QUIN) was applied [15]. This instrument evaluates experimental design and reports transparency against the following 12 criteria: aims, sample description, sampling technique, control group, methodological detail, consistency, outcome measures, outcome appropriateness, blinding, statistical analysis, results reporting, and study limitations. For in vivo components, the SYstematic Review Centre for Laboratory Animal Experimentation (SYRCLE) Risk of Bias tool was used [16]. It assesses methodological rigor against 10 criteria, including sequence generation, baseline similarity, allocation concealment, random housing, caregiver/investigator blinding, random outcome assessment, outcome assessor blinding, incomplete outcome data, selective reporting, and other potential sources of bias.

### 2.6. Data Synthesis

A narrative synthesis approach was adopted for this review [17], as the included studies were predominantly mechanistic and preclinical in nature and reported a range of heterogeneous outcomes. Most studies presented narrative findings or non-standardized quantitative measures, including changes in protein expression, proportion of CSC populations, colonosphere formation, tumor volume, and growth inhibition. Given this methodological and outcome heterogeneity, a meta-analytical approach was not feasible.

## 3. Results

**Figure 1:**
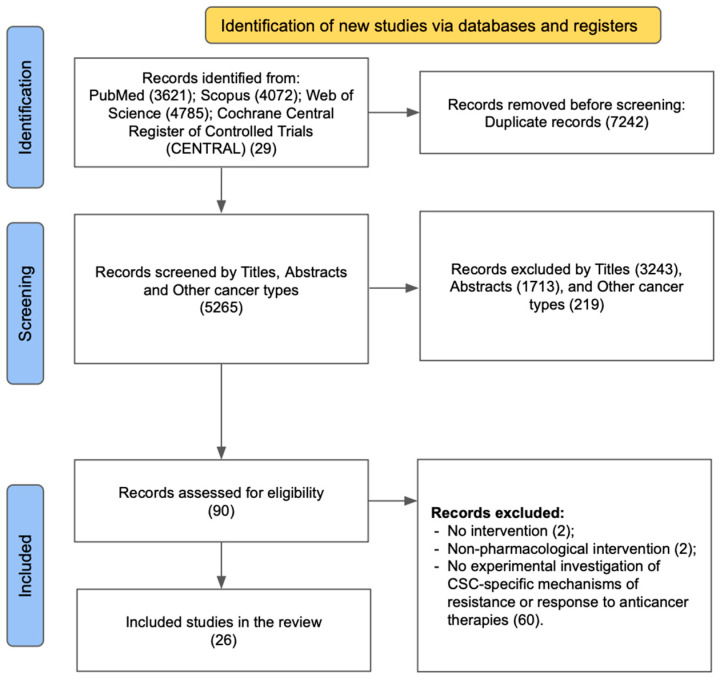
PRISMA flow diagram of the screening and selection process for this systematic review.

The initial search yielded 12,507 records. After removal of 7242 duplicates and exclusion of 64 records that did not meet the inclusion criteria, 26 studies were included in the final review (Figure 1). The included studies were published between 2015 and 2025 and originated from 12 different countries, including China [18,19,20,21,22,23,24,25], USA [22,26,27], the Republic of Korea [28,29], Italy [30,31,32,33,34,35,36], the Netherlands [33], Spain [34], Japan [37], India [38,39], Australia [40,41], France [41,42], Canada [42], and Taiwan [43].

All 26 shortlisted studies used in vitro experimental models to investigate the resistance mechanisms of CRC-SCs. These models predominantly used established human CRC-SC lines, including HCT116, HT-29, and SW480 [18,19,20,21,22,23,25,26,27,28,29,34,35,37,38,39,40,41,42,43], as well as patient-derived samples [22,24,30,31,32,33,36,37,39,41]. Furthermore, 20 out of 26 studies also incorporated in vivo experimental components, primarily using subcutaneous xenografts in immunocompromised mouse strains, including NOD/SCID, NSG, BALB/c, or nude mice [20,21,22,23,24,25,26,28,30,31,32,33,34,35,37,38,40,41,42,43]. For analytical purposes, the included studies were grouped according to the predominant signaling pathway mechanisms implicated in CSC-mediated therapeutic resistance. The overview of the included studies is presented in Table 1.

### 3.1. Risk of Bias Assessment

The QUIN assessment indicated that all included in vitro experiments were of moderate methodological quality. The domains most frequently rated as not specified were outcome assessor details, blinding, and operator details. In the SYRCLE assessment of in vivo experiments, there was a consistent pattern of unclear or high risk of bias across several methodological domains, particularly those related to allocation concealment, random housing, blinding of caregivers/investigators, random outcome assessment, and blinding of outcome assessors. The risk of bias assessment is presented in Supplementary Materials (Tables S1–S4).

### 3.2. Adaptive Signaling Pathways

Adaptive signaling plasticity was identified in 10 studies as a central mechanism underlying therapeutic resistance in CRC-SCs [18,21,24,27,29,30,32,33,34,40]. Across these studies, resistance was consistently linked to the capacity of CRC-SCs to activate compensatory signaling pathways following therapeutic exposure. In this context, inhibition of a single kinase, including BRAF, EGFR, AURKA, and related signaling components, frequently resulted in feedback activation of alternative survival pathways. This adaptive redundancy enabled CSC survival under therapeutic pressure and contributed to sustained tumor progression [18,21,24,27,29,30,32,33,34,40]. Collectively, these findings suggest that CRC-SCs rely on highly flexible, adaptive, and interconnected signaling networks that may limit the efficacy of therapeutic agents.

A prominent example of this adaptive behavior was evident in *BRAF*-mutant tumors, where inhibition of BRAF kinase with vemurafenib was insufficient to achieve sustained therapeutic response [18]. Studies using *BRAF*(V600E) mutant cell lines and xenograft models suggested that resistance is mediated by a feedback-driven upregulation of epidermal growth factor receptor (EGFR), which activates downstream MAPK signaling and maintains stemness-associated proliferative capacity [18]. In this setting, combined targeting with dabrafenib and cetuximab was required to reduce stemness markers, including CD44, Oct4, CBX-7, and EZH2, and to induce apoptosis, highlighting the limitations of single-node inhibition [18]. However, the signaling adaptability in CSCs extended beyond intrinsic feedback activation.

Adaptive signaling in CRC-SCs is further shaped by tumor microenvironment-mediated mechanisms. Prasetyani et al. showed that stromal-derived ligand NRG-1β activated ErbB-3/PI3K/AKT and ERK signaling in *BRAF*-mutant CSCs, thereby bypassing vemurafenib-mediated BRAF inhibition [33]. Pharmacological targeting of ErbB-3 receptors with the EV20 monoclonal antibody effectively disrupted this compensatory pathway and restored vemurafenib sensitivity, highlighting microenvironmental crosstalk in therapy resistance [33].

A comparable pattern of signaling reprogramming was observed in *RAS/RAF* wild-type models exposed to anti-EGFR therapy, including SW48 and C10 cell lines and patient-derived xenografts (PDX) [34]. In this context, AURKA-mediated phosphorylation of the transcription factor YAP1 at Ser397 promoted its stabilization and transcriptional activity, driving a stem-like phenotype [34]. This modification induced YAP1 upregulation, which promoted c-MET receptor expression as an alternative survival pathway [34]. Inhibition of AURKA with alisertib effectively disrupted this response and restored sensitivity to cetuximab, as evidenced by reduced levels of phosphorylated ERK and AKT, which are key downstream effectors of c-MET signaling [34].

Resistance to existing therapies was also associated with the adaptive reprogramming of downstream signaling pathways. Across MEK- and EGFR-targeted models, CRC-SCs did not remain dependent on the initially inhibited pathway, but instead reactivated multiple compensatory survival networks involving PI3K/AKT, JAK/STAT, Wnt, RAS/MEK/ERK, and PI3K/AKT/mTOR signaling axes [27,30]. Following cyclic MEK inhibition with trametinib, colorectal tumor spheroids acquired CSC-like phenotypes marked by increased CD166 and ALDH1A3 expression, alongside concurrent activation of PI3K/AKT, JAK/STAT, and Wnt pathways [27]. Notably, dual inhibition of MAPK/PI3K signaling was insufficient to fully suppress this adaptive state, whereas combined treatment with trametinib and mithramycin A more effectively attenuated CSC-associated phenotypes, feedback mechanisms, and invasive behavior [27]. A similar pattern of broad signaling reactivation was observed in cetuximab-resistant patient-derived CSCs, which restored activity across PI3K/AKT/mTOR, RAS/MEK/ERK, and JAK/STAT pathways following EGFR blockade, in contrast to cetuximab-sensitive cells, which exhibited coordinated pathway suppression [30]. Importantly, resistance in these models was not fully reversed by targeting individual compensatory pathways, suggesting that CRC-SCs rely on integrated signaling networks rather than one linear escape mechanism [27,30]. This was further supported by the enhanced efficacy of combination strategies, including trametinib with mithramycin A, which suppressed stemness, feedback mechanisms, EMT-related behavior, and invasiveness and by the synergy between cetuximab and PI3K/mTOR inhibition in resistant CSCs [27,30].

This adaptive logic also extended to CD44v6-positive colorectal cancer spheroid cells, which resisted BRAF-based combination therapy through compensatory PI3K/AKT activation [32]. Addition of a PI3K inhibitor to the BRAF/EGFR/HER2-targeted treatment transiently reduced CD44v6-positive cells, sphere formation, and xenograft growth; however, tumors regrew after treatment withdrawal [32]. This resistance was supported by elevated Myc levels, a transcription factor from the PI3K/AKT/mTOR axis [32]. Concomitant PI3K inhibition with taselisib, together with indirect Myc suppression via CDK inhibition, reduced cancer-associated fibroblast (CAF)-mediated protection, attenuated PI3K/AKT signaling activity and Myc expression, and inhibited xenograft tumor progression [32].

Beyond the major MAPK, PI3K/AKT, and receptor tyrosine kinase networks, several studies identified additional regulatory axes that supported CRC-SC resistance. Although these mechanisms were more heterogeneous, involving kinase activation, lipid-mediated signaling and microRNA-dependent pathway control, they converged on the preservation of stemness and treatment resistance. p21-activated kinase1 (PAK1) signaling represented one such kinase-dependent mechanism [40]. PAK1 contributed to 5-fluorouracil (5-FU) resistance by upregulating the expression of stem cell markers, including CD44, Nanog, and Bmi1, while PAK1 inhibition reduced CSC-associated features and increased 5-FU sensitivity [40]. MicroRNA-mediated regulation was also implicated in CRC-SC resistance: miR-199a/b promoted cisplatin resistance by targeting GSK3β and activating the Wnt/β-catenin/ABCG2 axis [24], while the transcription factor E2F7 promoted stemness by repressing miR-199b, thereby stabilizing the MAPK pathway via USP47 [21].

Collectively, these studies demonstrate that CRC-SCs employ redundant and highly flexible survival mechanisms under therapeutic pressure. Although the certainty of individual pathway-specific conclusions is limited by heterogeneity in treatment context, mutational background, and signaling readouts, adaptive signaling plasticity should be interpreted as a recurrent and well-established pattern in CRC-SC resistance. Rather than depending on a single dominant pathway, they dynamically rewire and switch between multiple signaling networks to preserve stemness and therapeutic resistance.

### 3.3. Epigenetic and Transcriptional Reprogramming

Therapeutic resistance in CRC-SCs was further linked to epigenetic changes and transcriptional mechanisms that preserve stem-like states, regulate drug metabolism, and modulate treatment susceptibility. Four studies examined these mechanisms, including nuclear receptor-driven transcriptional regulation, histone and protein methylation, and dormancy-associated cell-state control [35,37,41,42]. Although these studies focused on distinct regulatory layers, they converged on a shared principle: CRC-SCs evade therapy not only through activation of signaling pathways but also via epigenetic and transcriptional reprogramming, thereby reducing drug responsiveness [35,37,41,42].

One major mechanism involved transcriptional control of drug metabolism and efflux, specifically through the pregnane X receptor (PXR)-mediated transcriptional regulation [41]. In CSC-enriched spheroids, increased PXR activity was positively correlated with CSC marker expression and PXR target genes, including drug efflux transporters (ABCG2) and metabolic enzymes (CYP3A4) [41]. PXR inhibition reduced CSC enrichment, sphere-forming potential, chemoresistance, and post-chemotherapy tumor relapse in xenograft models [41]. These findings highlight transcriptional control of drug metabolism as a key determinant of CSC-mediated therapeutic resistance.

A related theme was that epigenetic regulators maintained CSC identity through the control of stemness-associated transcriptional programs. SMYD3-mediated methylation of c-Myc was identified as one such mechanism, in which SMYD3 interacted with c-Myc and methylated it at K158/K163, thereby supporting c-Myc transcriptional activity and the expression of stemness-associated target genes [35]. Pharmacological inhibition of SMYD3 with EM127 reduced tumorsphere formation, CSC marker expression, invasion, and tumor growth. EM127 also enhanced 5-FU-induced apoptosis [35]. Thus, these findings suggest that SMYD3 contributes to therapeutic resistance by sustaining c-Myc-dependent stemness programs, rather than primarily through modulation of drug efflux [35].

Histone methylation also contributed to CSC maintenance via the DAT-G9a axis. Vanoxerine, a dopamine transporter antagonist, suppressed CSC self-renewal by targeting a DAT-associated AKT/Nur77/G9a pathway [42]. This was associated with decreased neoplastic stemness, reduced tumor initiation, and increased tumor immune-cell infiltration, including type-1 IFN-related immune activation, in preclinical models [42]. Together, these findings suggest that methylation-dependent regulation sustains the CRC-SC phenotype at multiple regulatory levels, including both direct modulation of oncogenic transcriptional programs and chromatin-associated remodeling. Furthermore, resistance was also linked to dormancy-associated cell-state regulation [37]. FBXW7 facilitated survival by maintaining CSCs in a dormant, non-dividing state, thereby reducing their susceptibility to chemotherapy. Silencing FBXW7 promoted cell-cycle reentry and increased treatment susceptibility [37]. Unlike mechanisms centered on drug metabolism or stemness-associated transcriptional programs, this strategy confers resistance primarily through preservation of a therapy-insensitive cellular state.

Together, these studies indicate that epigenetic and transcriptional mechanisms contribute to CRC-SC resistance by preserving stem-like states, regulating drug metabolism, and modulating treatment susceptibility. The evidence was strengthened using patient-derived models, organoids, xenografts, and pharmacological perturbation experiments. However, the studies differed in targets, CSC-enrichment strategies, and therapeutic contexts. Therefore, although these findings support epigenetic and transcriptional reprogramming as an important contributor to CRC-SC resistance, the relevance of each finding across broader CRC molecular subtypes and treatment strategies remains to be established.

### 3.4. DNA Damage Response and Apoptosis

CRC-SCs were shown to resist cytotoxic therapies through enhanced DNA damage repair, checkpoint activation, and evasion of apoptosis. Across four identified studies, CRC-SCs survived cytotoxic stress through activation of checkpoint kinases, homologous recombination, mismatch-repair-related mechanisms, and suppression of death receptor-mediated apoptosis [26,31,36,39].

One key component of this response was the checkpoint kinase CHK1, which regulates cellular responses to replication stress and DNA damage [31]. When activated, CHK1 helps preserve genomic integrity by delaying cell-cycle progression and allowing DNA repair before mitosis begins. In one study, treating CSCs with the synthetic alkaloid NORA234 induced genotoxic stress, but stem cells survived by upregulating CHK1 [31]. The addition of a CHK1 inhibitor prevented this upregulation, leading to a decrease in CD44v6+ stem cells, which are associated with constitutive Wnt pathway activation [31]. The importance of CHK1-mediated protection was further supported by Mattiello et al., who showed that the CHK1 inhibitor had limited efficacy when used alone; however, combining it with inhibitors of homologous recombination, such as RAD51 or DNA break-sensing MRE11, improved the outcome [36]. This combination increased replication stress, disrupted checkpoint control, and promoted premature mitotic entry, thereby eradicating CRC-SCs through caspase-dependent apoptosis [36]. These findings indicate that DNA damage tolerance in CRC-SCs involves coordinated checkpoint and repair mechanisms rather than a single protective pathway.

Alongside checkpoint modulation, CSCs used mismatch-repair (MMR)-associated mechanisms to survive under 5-FU therapy. The PARP inhibitor veliparib facilitated 5-FU cytotoxicity by inhibiting PARP1-mediated PARylation and disrupting the interaction between PARP1 and MSH6, a mismatch repair protein. This led to S-phase arrest and apoptosis [39]. Despite DNA damage, CSCs can still proliferate by interfering with death receptor-mediated apoptotic pathways. For example, Huang et al. reported that CD133+ colon CSCs evade Fas-mediated apoptosis [26]. This effect was mediated by overexpression of Fas-associated phosphatase 1 (Fap1), which neutralized Fas-dependent apoptotic signaling. Inhibition of Fap1 restored Fas-dependent apoptosis and increased oxaliplatin sensitivity in this stem-like compartment [26].

These findings indicate that CRC-SCs resist cytotoxic therapy through two complementary strategies: mitigating the consequences of DNA damage and suppressing apoptosis. This dual protection may allow CSCs to survive genotoxic stress and persist after treatment. However, the relevance of these findings is limited to study-specific constraints, including reliance on selected CSC marker-defined populations, limited assessment across broader CSC phenotypes, and incomplete validation in metastatic models. Therefore, DNA damage response and apoptotic regulation appear to represent an important mechanistic vulnerability, but their relevance remains to be established in clinically diverse CRC-SC populations.

### 3.5. Tumor Microenvironment and Immune Evasion

TME resistance was described in two studies [19,20], both of which showed that CRC-SC survival was shaped by extrinsic cytokine, immune, and metabolic signals rather than by cell-intrinsic mechanisms alone. Although these studies examined different therapeutic strategies, they shared a common concept: CRC-SCs can exploit microenvironmental signals to preserve stemness, evade immune-mediated elimination, and reduce treatment sensitivity.

In bevacizumab-resistant CRC-SCs, this protection was mediated through the activation of the IL-22/STAT3 axis. Resistant CD133/EpCAM-positive CSCs showed increased expression of IL-22, IL-22R, GP130, and STAT3, along with enhanced tumorigenicity in xenograft models. Functionally, blocking IL-22 reduced stemness markers, including Oct4, Sox2, EpCAM, and CD133, as well as angiogenic mediators such as VEGF and VEGFR [20]. Moreover, when combined with bevacizumab, IL-22 inhibition further suppressed xenograft tumor growth, suggesting that IL-22/STAT3 supports both CSC maintenance and resistance to antiangiogenic agents [20]. While this mechanism demonstrates how cytokine signaling can sustain CSC features and antiangiogenic resistance, the second study extended the role of the tumor microenvironment to immune escape under hypoxic conditions. Under these conditions, co-culturing with NK cells induced upregulation of the FADS1/DDA axis in CRC-SCs, suppressing NK cell cytotoxicity and reducing IFN-γ/TNF-α secretion [19]. The same co-culture conditions were also associated with increased IL-6 secretion, suggesting broader cytokine dysregulation during CRC-SC immune evasion [19].

Together, these studies show that CRC-SC resistance is shaped not only by cell-intrinsic programs but also by hypoxic, immune, and cytokine-mediated signals within the surrounding microenvironment. Nevertheless, the strength of evidence differed between studies: the IL-22/STAT3 mechanism reported by Qin et al. was supported by both in vitro and in vivo models [20], whereas the FADS1/DDA mechanism was derived mainly from an in vitro hypoxic co-culture model [19]. Accordingly, TME-mediated resistance should be interpreted as a biologically plausible but still hypothesis-generating mechanism requiring further confirmation in standardized in vivo models, patient-derived models, and clinically relevant samples.

### 3.6. Natural Compound-Derived Interventions

Six studies evaluated natural compound-derived agents as potential sensitizing agents against CRC-SCs [22,23,25,28,38,43]. Across these studies, the agents affected both intrinsic CSC-associated pathways and extrinsic microenvironmental processes, including Wnt/β-catenin signaling, Notch signaling, angiogenesis, exosomal communication, macrophage-associated inflammation, and EMT-related pathways.

One study reported that 5-FU exposure enriched CSC-like populations. Metformin, in combination with the Wnt inhibitor ICG-001, reduced this resistance by inducing apoptosis and autophagy [22]. This combination inhibited Wnt/β-catenin signaling and decreased the expression of CSC-associated core markers, including CD44 and Oct4.

Sulforaphane (SFN), an isothiocyanate phytochemical found in cruciferous vegetables, was evaluated in two studies. Two independent studies suggested that SFN suppressed CRC-SC properties by modulating stemness-associated regulatory axes [23,25]. First, SFN was found to downregulate ΔNp63α expression, which is responsible for the expression of key stemness transcription factors, including Nanog, Oct4, and Sox2 [23]. This was associated with reduced tumorsphere formation and decreased expression of CSC markers, including CD133, CD44, and Lgr5. Furthermore, SFN also acted as an inhibitor of the ZNF217/Notch pathway. ZNF217 usually activates the Notch signaling pathway to maintain stemness, but SFN administration decreased the levels of ZNF217 and Notch1 proteins [25].

Orientin, a natural flavonoid, was investigated in combination with 5-FU. 5-FU was associated with angiogenesis and systemic toxicity, whereas orientin attenuated these effects. Mechanistically, orientin reduced NF-κB expression and HIF1*α* activity, disrupting the NF-κB/HIF1*α*/VEGFA axis and downregulating VEGF production, thus inhibiting angiogenesis, which is necessary for tumor growth and development [38]. In vivo models indicated that the combination of orientin and 5-FU reduced CD44+/CD133+ CSC populations, tumor volume, and 5-FU-associated hepatotoxicity and nephrotoxicity [38].

Ovatodiolide, a small-molecule phytochemical, was shown to target exosome-mediated communication in colorectal tumorspheres. Colorectal tumorspheres released exosomes carrying oncogenic signals that supported CSC properties and TME [43]. Ovatodiolide prevented this trafficking, thereby decreasing exosomal transport of β-catenin, p-STAT3, IL-6, TGF-β1, and miR-1246 [43]. Through this mechanism, ovatodiolide reduced the conversion of normal fibroblasts into CAFs and limited M2 macrophage polarization [43]. Likewise, ovatodiolide was shown to decrease CSC colony formation, migration, and 5-FU resistance, potentially through interference with β-catenin/STAT3/miR-1246-mediated exosomal signaling [43].

A marine omega-3 fatty acid-derived compound, diHEP-DPA (7S,15R-dihydroxy-16S,17S-epoxy-docosapentaenoic acid), was investigated in the context of 5-FU-associated macrophage-mediated chemoresistance [28]. Prolonged 5-FU treatment was associated with increased M2-like tumor-associated macrophage (TAM) features and secretion of growth factors, including VEGF and IL-6 [28]. These factors may contribute to chemoresistance. DiHEP-DPA was suggested to suppress M2-like TAM-associated signaling by inhibiting the NF-κB signaling pathway. It also interfered with epithelial–mesenchymal transition (EMT) through inhibition of the STAT3 signaling pathway [28].

Collectively, these studies suggest that natural compound-derived agents may reduce CRC-SC-associated resistance by targeting multiple signaling, metabolic, angiogenic, and microenvironmental mechanisms. However, the strength of the evidence should be interpreted cautiously because the studies were heterogeneous in design, model systems, tested agents, and outcome measures. Together with the moderate methodological quality of the in vitro studies and unclear or high risk-of-bias concerns in several in vivo domains, these limitations indicate that natural compound-derived agents should be considered promising preclinical sensitizers rather than clinically established therapeutic agents.

## 4. Discussion

The present systematic review synthesized preclinical evidence on molecular mechanisms underlying modern therapy resistance in CRC-SCs, drawing on 26 included in vitro and/or in vivo studies. Although the included studies were heterogeneous in design and reported outcomes, the findings collectively indicate that therapeutic resistance in CRC-SCs is not driven by a single dominant mechanism but rather by an adaptive, multilayered regulatory network. Key contributors include adaptive signaling pathways, epigenetic reprogramming, enhanced DNA damage response, and interactions with the tumor microenvironment, which together establish a coordinated survival system under therapeutic stress.

A central observation from the included preclinical evidence was that CRC-SC-associated resistance appears to be strongly linked to cellular plasticity. Instead of relying on fixed resistance programs, CRC-SCs may dynamically adapt to therapeutic pressure through the activation of compensatory signaling networks. The involvement of signaling pathways in CSC-mediated resistance has been extensively described in previous studies [44,45,46]. Consistent with this literature, the present systematic review identified the MAPK, JAK/STAT, PI3K/AKT/mTOR, and Wnt/β-catenin pathways as key regulators of tumorigenesis and CSC-associated resistance mechanisms. Importantly, these pathways do not function independently but are interconnected through extensive crosstalk, forming a coordinated signaling network. This interdependence helps explain why inhibition of a single pathway is frequently insufficient to produce durable therapeutic effects.

In addition, alterations in *BRAF* and *RAS* contribute significantly to therapeutic resistance in CRC-SCs. This form of resistance may be partially overcome through combination treatment strategies, for example, *BRAF*(V600E)-mutant CRC often exhibits limited responsiveness to BRAF inhibition due to feedback activation of EGFR signaling, which sustains downstream proliferative and survival pathways. However, combined inhibition of BRAF and EGFR has been shown to disrupt this compensatory axis, thereby enhancing cytotoxicity and promoting cell-cycle arrest [18]. These findings further highlight the molecular heterogeneity underlying resistance in CRC-SCs. From a translational perspective, they suggest that genetic and pathway-based profiling may be useful in future studies to define molecular contexts in which specific CSC-associated resistance mechanisms are relevant. However, because the evidence synthesized here is preclinical, the use of these mechanisms for patient stratification or treatment selection requires validation in clinically relevant patient cohorts and prospective studies.

Importantly, the findings of this review indicate that CRC-SC resistance is not solely determined by cell-intrinsic mechanisms but is also substantially reinforced by the TME. Hypoxic conditions within the TME, together with dynamic interactions among stromal and immune cells, promote and maintain stem-like phenotypes [47,48]. In particular, hypoxic environments have been associated with immune evasion through activation of the FADS1/DDA axis. Although a therapeutic agent such as bevacizumab, a VEGF inhibitor, has been reported to improve chemosensitivity [49], its efficacy may be attenuated by microenvironmental signals. For example, IL-22, secreted from the microenvironment, can activate STAT3 signaling in CRC-SCs, thereby reducing sensitivity to anti-VEGF therapy. Conversely, preclinical findings suggest that certain therapeutic combinations may counteract these resistance mechanisms. For instance, the natural compound orientin, when combined with 5-FU, has been shown to inhibit angiogenesis in CSCs through the suppression of the HIF1α/VEGFA axis.

Conventional anticancer therapies effectively eliminate the bulk of tumor cells; however, a subpopulation of CSCs often persists and is implicated in tumor relapse [50]. Notably, certain treatments, including 5-FU and BRAF inhibitors, have been reported to paradoxically enrich the CSC population [28,38]. Similar observations have been described in gastric cancer, where 5-FU exposure was associated with increased stemness, tumorigenicity, and self-renewal [51]. In parallel, studies in breast cancer have demonstrated that chemotherapy can lead to the elevated expression of CSC markers in breast cancer stem cells [50]. These findings suggest that conventional therapies may inadvertently select for or induce stem-like phenotypes, thereby contributing to disease recurrence. These observations provide a rationale for further preclinical evaluation of combination strategies designed to target both differentiated tumor cells and CSC populations. However, whether such approaches reduce relapse risk in patients remains to be established clinically.

To the best of our knowledge, this is the first systematic review of preclinical evidence on molecular mechanisms of resistance in CRC-SCs. Inclusion of both in vivo and in vitro models, as well as patient-derived samples in selected studies, enabled comparison of resistance mechanisms across different experimental systems, thereby enhancing the robustness and translational relevance of the synthesis. However, this approach had several limitations. Firstly, although all included studies incorporated in vitro experiments, and 20 employed in vivo models, these systems cannot fully recapitulate the complexity of human tumor biology. Consequently, the mechanisms identified in this review should be regarded as preclinical evidence rather than direct clinical proof and will require further validation in clinical datasets. Secondly, some studies reported molecular findings derived from relatively small cohorts of CRC patients, which may limit the generalizability of the results to broader populations. Thirdly, due to heterogeneity in study designs and the limited methodological quality of the included studies, the findings of the current review should be interpreted with caution.

The findings of this review have several potential translational implications. The preclinical evidence suggests that CRC-SC-associated resistance is unlikely to be explained by a single pathway, as these cells appear to rely on multiple interconnected signaling networks. Accordingly, future translational studies may benefit from evaluating CSC-associated biomarkers and functional CSC phenotypes alongside conventional measures of treatment response. Such work could help determine whether CSC-related mechanisms are relevant to patient stratification. In addition, natural compound-derived agents may warrant further preclinical studies as potential sensitizers, particularly where they appear to have an effect on multiple CSC-associated resistance pathways. However, their therapeutic relevance requires investigation in standardized models and, ultimately, clinical studies.

## 5. Conclusions

CRC-SC-mediated resistance appears to arise from a coordinated network of adaptive mechanisms rather than a single dominant pathway. Across the included studies, the resistance to therapeutic stress was supported by signaling plasticity, epigenetic and transcriptional reprogramming, enhanced DNA damage response, evasion of apoptosis, and tumor microenvironment-mediated protection. However, the available evidence remains predominantly preclinical and heterogeneous, with most findings derived from in vivo, in vitro, and selected patient-derived models. Therefore, these mechanisms should be interpreted as biologically plausible contributors to resistance, but not as clinically validated determinants of treatment response. Future studies should prioritize clinically relevant models and combination approaches that evaluate both intrinsic CSC plasticity and extrinsic components of the tumor microenvironment. Such strategies may help clarify the translational relevance of CRC-SC resistance and develop more durable therapeutic agents.

## Figures and Tables

**Table 1:** The overview of included studies.

First Author	Country	Aim	Intervention	Experimental Strategy	Main Finding
Wu et al., 2018 [18]	China	Test whether combined BRAF/EGFR inhibition improves cytotoxicity and reduces stemness in *BRAF*(V600E) CRC	Dabrafenib + cetuximab as the main combination	Combination treatment in *BRAF*(V600E) mutant cell lines and xenografts	Combined inhibition increased cytotoxicity and decreased stem-cell capacities compared to single agents
Geng et al., 2024 [19]	China	Determine how CRC-SCs evade natural killer (NK) cell-mediated killing under varying oxygen conditions, and to identify metabolic and genetic pathways responsible for this evasion.	Exposure of CRC-SCs cells to NK cells under hypoxia; FADS1 siRNA in CCSCs ± exogenous DDA in CSC-NK hypoxia co-culture	Co-culture of CSCs and NK cells under normoxic and hypoxic conditions	CSCs under hypoxia reduced NK cytotoxicity and decreased IFN-γ and TNF-α, and increased IL-6; FADS1 → DDA axis mediated immune evasion
Qin et al., 2023 [20]	China	Investigate how colorectal CSCs contribute to bevacizumab resistance, focusing on the IL-22/STAT3 signaling pathway	IL-22 knockdown (shRNA) in bevacizumab-resistant CRC-SCs + bevacizumab	Co-culture and knockdown in bevacizumab-resistant cells	Bevacizumab-resistant CRC-SCs showed increased activation of the IL-22/STAT3 pathway with higher expression of IL-22, IL-22R, and STAT3. Blocking IL-22 reduced stemness markers CD133, EpCAM, Oct4 and Sox2, tumor growth and restored sensitivity to bevacizumab
Guo et al., 2020 [21]	China	Determine how E2F7 regulates colon CSC activity via miR-199b/USP47	E2F7–miR-199b–USP47 axis	Knockdown/overexpression of E2F7, miR-199b and USP47 in cell lines	E2F7 promoted tumor stem cell activity by miR-199b suppressing, upregulating USP47, and stabilizing MAPK signaling
Roy et al., 2022 [22]	USA, China	Test whether the combination of metformin and Wnt inhibitor can overcome CSC-mediated 5-FU resistance	Metformin + ICG-001, alone or combined	Co-treatment of 5-FU-resistant cells with metformin and ICG-001	A combination of metformin + ICG-001 reduced CSC markers, spheroid formation, and promoted apoptosis
Chen et al., 2022 [23]	China	Define ΔNp63α role in CSC regulation and sulforaphane (SFN) response	SFN; ΔNp63α overexpression/knockdown	Treatment of sphere-forming cells with SFN; ΔNp63α overexpression/knockdown and testing SFN effects in vitro and in vivo	ΔNp63α promoted CSC features through the activation of Nanog, Oct4, and Sox2 transcription. SFN was able to inhibit this link
Chen et al., 2017 [24]	China	Determine whether miR-199a/b is involved in cisplatin resistance in ALDHA1+ CSCs and through which pathway	Cisplatin; miR-199a/b antagomir; ABCG2 knockdown	ALDHA1+ CSCs were isolated and then compared with ALDHA1- cells. They assessed sphere formation, tumor initiation, cisplatin response, miR-199a/b manipulation, Wnt inhibition, ABCG2 knockdown and xenografts	miR-199a/b promoted cisplatin resistance in ALDHA1+ CRC-SCs via GSK3β suppression, Wnt/β-catenin activation, and ABCG2-linked drug efflux
Wang et al., 2024 [25]	China	To identify whether ZNF217 activates Notch signaling to promote CSC, and whether this mediates SFN response	SFN; ZNF217 overexpression/knockdown; Notch1 knockdown	Administration of SFN to CRC cell lines and xenografts, and evaluation of ZNF217/Notch signaling	ZNF217 activated Notch signaling, thereby increasing CSC markers and sphere formation. SFN suppressed CSC features via inhibition of the ZNF217/Notch1 axis
Huang et al., 2018 [26]	USA	To determine whether Fap1 mediates apoptosis resistance in colon CSCs and whether Fap1 inhibition enhances oxaliplatin response	FAP1 inhibition + oxaliplatin	Treatment of CSCs cells with Fap1 inhibition and oxaliplatin	CSCs expressed high levels of Fap1 and were resistant to Fas- and oxaliplatin-induced apoptosis. Fap1 inhibition facilitated apoptosis in colon CSCs and enhanced oxaliplatin effects
Lamichhane et al., 2022 [27]	USA	Determine whether targeting CSCs can prevent resistance to MEK inhibition	Cyclic MEK inhibition with trametinib/selumetinib/PD0325901; resistance prevention with trametinib + mithramycin A	Combination treatment with the MEK inhibitor	MEK inhibition induced CSC markers CD166/ALDH1A3, compensatory PI3K/AKT, JAK/STAT, and Wnt signaling, and invasion. Trametinib + mithramycin A suppressed CSC-linked resistance and invasion
Su et al., 2023 [28]	Republic of Korea	Test whether diHEP-DPA can overcome 5-FU resistance by suppressing TAMs, CSCs, and EMT	diHEP-DPA alone and combined with 5-FU	Administration of diHEP-DPA, 5-FU, and combination	diHEP-DPA reduced TAM infiltration, CSC enrichment, suppressed β-catenin, and inhibited NF-κB/STAT3 signaling and EMT markers
Kim et al., 2025 [29]	Republic of Korea	Determine whether PF-543, a SPHK1 inhibitor can overcome TRAIL resistance and identify the mechanism involved	PF-543 + TRAIL	Administration of PF-543, TRAIL, and combination in TRAIL-resistant cells	PF-543 sensitized TRAIL-resistant CRC cells via SPHK1/S1PR1/STAT3 pathway, activating apoptosis and reducing stemness/EMT traits
De Angelis et al., 2016 [30]	Italy	Identify molecular determinants of therapy resistance using CSC models	Cetuximab; irinotecan; cetuximab + irinotecan	Established and validated CSC spheroid lines, characterized them genomically and proteomically and used them in drug response assays	Cetuximab resistance in CRC-SCs was associated with persistent activation of PI3K/AKT/mTOR, RAS/MEK/ERK and JAK/STAT pathways despite EGFR blockade
Di Franco et al., 2021 [31]	Italy	Test whether NORA234 can target CSCs and whether CHK1-mediated DNA damage response can overcome adaptive resistance to NORA234	NORA234 (nortopsentin analog); CHK1 inhibition	Treatment of patient-derived spheres with nortopsentin alone or with CHK1 inhibitor	A combination of NORA234 and CHK1 inhibition depleted resistant CSCs to nortopsentin, increased apoptosis
Gaggianesi et al., 2022 [32]	Italy	Evaluate whether combined inhibition of Myc transcription and PI3K activity can overcome resistance in colorectal CSCs	Dual Myc-transcription + PI3K targeting with dinaciclib and taselisib	Drug combination in CSCs and patient-derived xenografts	Dual inhibition effectively targeted CSCs, reducing clonogenicity, viability PI3K/Akt activity and Myc expression regardless of mutational background or Myc
Prasetyanti et al., 2015 [33]	Italy, The Netherlands	Analyze the role of NRG-1β/ErbB-3 signaling in growth and promotion of vemurafenib resistance in *BRAF*-V600E colon CSCs, and whether ErbB-3 inhibition can overcome this resistance	Vemurafenib treatment in BRAF-V600E colon CSCs; ErbB-3 knockdown or EV20 antibody inhibition	Treatment of *BRAF*-mutant CSCs with anti-ErbB-3 antibody	NRG-1β activated ErbB-3 in *BRAF*-V600E colon CSCs, which restored AKT/ERK signaling and promoted resistance to vemurafenib; blocking ErbB-3 reduced growth and restored vemurafenib sensitivity
Rio-Vilariño et al., 2024 [34]	Italy, Spain	Test whether AURKA/YAP1 axis contributes to cetuximab resistance and CSC-like features, and whether AURKA inhibition can restore sensitivity	Cetuximab + alisertib (AURKA inhibitor)	Treatment of cetuximab-resistant cells with AURKA inhibitor	The cell lines showed increased AURKA-mediated Yap1 Ser397 phosphorylation, which promoted cetuximab resistance. Inhibition of AURKA with alisertib reduced CSC features and restored sensitivity to cetuximab
Lepore Signorile et al., 2025 [35]	Italy	Determine whether SMYD3 regulates CSCs through c-Myc, and whether inhibition of SMYD3/c-Myc axis suppresses CSCs	EM127 (SMYD3 inhibitor), SMYD3 knockout, 5-FU combined with EM127	Treatment of cells and patient-derived xenografts with EM127 alone, in combination with 5-FU	SMYD3 regulated c-Myc activity; Its inhibition reduced self-renewal, tumorigenicity and enhanced 5-FU sensitivity
Mattiello et al., 2021 [36]	Italy	Determine DNA damage response targets that can overcome CRC-SC resistance to CHK1 inhibition, and test whether targeting MRE11 or RAD51 sensitized CSCs to CHK1/2 inhibitor	Prexasertib (CHK1/2 inhibitor) + mirin (MRE11)/B02 (RAD51) inhibitors	Combination of CHK1 inhibitor with MRE11 or RAD51 inhibitors in CSC	Targeting MRE11 or RAD51 sensitized CRC-SCs to CHK1 inhibition, forcing damaged cells into apoptosis
Honma et al., 2019 [37]	Japan	Define how FBXW7 relates to chemoresistance in CRC-SCs	FBXW7 knockdown; 5-FU, oxaliplatin, and irinotecan	Analysis of FBXW7 expression in patient-derived samples and cell lines; knockdown	FBXW7-high associated with poor response; FBXW7 supported chemoresistance via cell-cycle arrest, and knockdown increased drug sensitivity
Ghosh et al., 2025 [38]	India	Determine whether orientin can reduce 5-FU-induced CRC-SCs enrichment and suppress angiogenesis via HIF1α/VEGFA axis	Orientin + 5-FU	Compared orientin, 5-FU and combination treatment, assessing CSC markers, oxidative stress, HIF1α/VEGFA expression, and tumor growth	Combination reduced CD44+/CD133+ CSCs, inhibited NF-κB/HIF1α/VEGFA signaling, angiogenesis, stem genes, reduction of 5-FU toxicity and tumor volume
Paul et al., 2023 [39]	India	Determine whether PARP inhibition can enhance 5-FU cytotoxicity in CRC-SCs by disrupting PARP1-MSH6-mediated mismatch repair pathway	PARP inhibitor (veliparib/ABT-888) + 5-FU	Combination with 5-FU	Veliparib sensitized CRC-SCs to 5-FU by disrupting the PARP1-MSH6-mediated pathway, thereby promoting DNA damage, apoptosis and tumor growth inhibition
Huynh et al., 2016 [40]	Australia	Test how PAK1 relates to CSC marker upregulation and 5-FU resistance	PAK1 inhibitor; 5-FU	Treatment of 5-FU-resistant cells with the PAK1 inhibitor	PAK1 activity increased expression of CSC markers, enhanced sphere formation, tumor growth and resistance to 5-FU; inhibition reversed these features
Planque et al., 2016 [41]	France, Australia	Test whether Pregnane X Receptor (PXR) promotes colon CSC chemoresistance, self-renewal and relapse	5-FU, leucovorin, irinotecan; PXR knockdown	shRNA knockdown of PXR in CSCs; treatment with standard chemotherapy	PXR knockdown sensitized CSCs to chemotherapy and delayed relapse
Bergin et al., 2024 [42]	Canada, France	Test whether DAT-G9a axis maintains CSCs and can be drug-targeted	Vanoxerine (dopamine transporter antagonist); DAT/G9a-axis modulation	Vanoxerine treatment and DAT/G9a-axis modulation assessed suppression of colon CSC self-renewal, tumor initiation, and immune evasion	Vanoxerine inhibited colon CSCs self-renewal and tumor initiation by suppressing DAT-AKT/Nur77/G9a signaling, while also promoting IFN-related immune activation
Huang et al., 2020 [43]	Taiwan	Determine whether Ovatodiolide can inhibit colon CSCs by disrupting CSC-derived exosomal signaling involved in 5-FU resistance, stemness, and TME	Ovatodiolide, 5-FU	Treatment of tumorspheres, isolation of exosomes, and assessment of stromal transformation with Ovatodiolide, 5-FU or both	Ovatodiolide impaired tumorsphere-derived exosomal β-catenin/STAT3/miR-1246 signaling, thereby decreasing 5-FU resistance, CAF and M2 TAM transformation, and tumor growth

## Data Availability

No new data were created or analyzed in this study. Data sharing is not applicable to this article.

## Supplementary Materials

The following supporting information can be downloaded at: https://www.mdpi.com/article/10.3390/ijms27146285/s1.

## Abbreviations

The following abbreviations are used in this manuscript:

5-FU	5-fluorouracil
ABCG2	ATP-binding cassette subfamily G member 2
AKT	Protein kinase B
ALDHA3	Aldehyde dehydrogenase 1 family member A3
AURKA	Aurora kinase A
Bmi1	B lymphoma Mo-MLV insertion region 1 homolog
BRAF	serine/threonine-protein kinase B-Raf
BRAF (V600E)	BRAF valine-to-glutamate substitution at codon 600
c-MET	Mesenchymal–epithelial transition factor
CAF	Cancer-associated fibroblast
CBX-7	Chromobox protein homolog 7
CDK	Cyclin-dependent kinase
CHK1	Checkpoint kinase 1
CRC	Colorectal cancer
CSC	Cancer stem cell
CRC-SCs	Colorectal cancer stem cells
DAT	Dopamine transporter
DHA	Docosahexaenoic acid
diHEP-DPA	7S,15R-dihydroxy-16S,17S-epoxy-docosapentaenoic acid
EGFR	Epidermal growth factor receptor
EMT	Epithelial–mesenchymal transition
ErbB-3	Erb-b2 receptor tyrosine kinase 3
ERK	Extracellular signal-regulated kinase
EZH2	Enhancer of zeste homolog 2
Fap1	Fas-associated phosphatase 1
GSK31β	glycogen synthase kinase 3 beta
IL-6	Interleukin 6
IL-22	Interleukin 22
JAK	Janus kinase
MAPK	Mitogen-activated protein kinase
MEK	Mitogen-activated protein kinase kinase
MMR	Mismatch repair
miR	MicroRNA
mTOR	Mechanistic target of rapamycin
NK	Natural killer
NRG-1β	Neuregulin-1 beta
Oct4	Octamer-binding transcription factor 4
PAK1	P-21 activated kinase 1
PDX	Patient-derived xenograft
PI3K	Phosphoinositide 3-kinase
RPPA	Reverse phase protein array
PRISMA	Preferred Reporting Items for Systematic Reviews and Meta-Analyses
PXR	Pregnane X receptor
QUIN	Quality Assessment Tool for In Vitro Studies
Ras	Rat sarcoma virus
S1PR1	Sphingosine-1-phosphate receptor 1
SFN	Sulforaphane
SPHK1	Sphingosine kinase 1
STAT3	Signal transducer and activator of transcription 3
STAT	Signal transducer and activator of transcription
SYRCLE	Systematic Review Centre for Laboratory animal Experimentation
TAM	Tumor-associated macrophages
TME	Tumor microenvironment
TRAIL	Tumor necrosis factor-related apoptosis-inducing ligand
USP47	Ubiquitin-specific peptidase 47
VEGF	Vascular endothelial growth factor
Wnt	Wingless signaling pathway
YAP1	Yes-associated protein 1
ZNF217	Zinc finger protein 217
