# Supplementary material for: Resistance of Colorectal Cancer Stem Cells to Modern Therapies: A Systematic Review
Source: Int J Mol Sci. 2026 Jul 15;27(14):6285. doi: 10.3390/ijms27146285 (PMC13409747; doi:10.3390/ijms27146285)
Supplement: Supplementary file 1 [file ijms-27-06285-s001.zip › File S1.pdf]

File S1. Search Terms.

Cancer* OR Breast OR Colorectal OR Lung OR Neoplasm* OR Tumor* OR Malignancy OR Carcinoma

AND

“Stem Cell*” OR “Cancer Stem Cell*” OR CSC OR “Tumor-initiating cell*” OR “Neoplastic stem cell*” OR “Anticancer cell*”

AND

“Therapy resistance” OR “Drug resistance” OR “Treatment resistance” OR Resistance OR Inhibit* OR Block* OR Mediate* OR “Car-T” OR "Immunotherap*" OR Therap* OR “Natural Killer Cell*”
