# Supplementary material for: Resistance of Colorectal Cancer Stem Cells to Modern Therapies: A Systematic Review
Source: Int J Mol Sci. 2026 Jul 15;27(14):6285. doi: 10.3390/ijms27146285 (PMC13409747; doi:10.3390/ijms27146285)
Supplement: Supplementary file 1 [file ijms-27-06285-s001.zip › File S2.pdf]

File S2. Search Strategy.

a) PubMed

#5			Search: #1 AND #2 AND #3 Filters: from 2015 - 2025 Sort by: Publication Date	3,621	14:31:48
#4			Search: #1 AND #2 AND #3 Sort by: Publication Date	4,944	14:31:23
#3			Search: "Therapy resistance"[Title] OR "Drug resistance"[Title] OR "Treatment resistance"[Title] OR Resistance[Title] OR Inhibit*[Title] OR Block*[Title] OR Mediate*[Title] OR "Car-T"[Title] OR "Immunotherap*"[Title] OR Therap*[Title] OR "Natural Killer Cell*"[Title] Sort by: Publication Date	2,552,197	14:30:59
#2			Search: "Stem Cell*"[Title] OR "Cancer Stem Cell*"[Title] OR CSC[Title] OR "Tumor-initiating cell*"[Title] OR "Neoplastic stem cell*"[Title] OR "Anticancer cell*"[Title] Sort by: Publication Date	189,055	14:30:44
#1			Search: Cancer*[Title] OR Breast[Title] OR Colorectal[Title] OR Lung[Title] OR Neoplasm*[Title] OR Tumor*[Title] OR Malignancy[Title] OR Carcinoma[Title] Sort by: Publication Date	2,691,862	14:30:26

b) Scopus

TITLE (Cancer* OR Breast OR Colorectal OR Lung OR Neoplasm* OR Tumor* OR Malignancy OR Carcinoma) AND TITLE ("Stem Cell*" OR "Cancer Stem Cell*" OR CSC OR "Tumor-initiating cell*" OR "Neoplastic stem cell*" OR "Anticancer cell*") AND TITLE ("Therapy resistance" OR "Drug resistance" OR "Treatment resistance" OR Resistance OR Inhibit* OR Block* OR Mediate* OR "Car-T" OR "Immunotherap*" OR Therap* OR "Natural Killer Cell*")

5,664 documents found

TITLE (Cancer* OR Breast OR Colorectal OR Lung OR Neoplasm* OR Tumor* OR Malignancy OR Carcinoma) AND TITLE ("Stem Cell*" OR "Cancer Stem Cell*" OR CSC OR "Tumor-initiating cell*" OR "Neoplastic stem cell*" OR "Anticancer cell*") AND TITLE ("Therapy resistance" OR "Drug resistance" OR "Treatment resistance" OR Resistance OR Inhibit* OR Block* OR Mediate* OR "Car-T" OR "Immunotherap*" OR Therap* OR "Natural Killer Cell*") AND PUBYEAR > 2014 AND PUBYEAR < 2026

4,072 documents found

TITLE (Cancer* OR Breast OR Colorectal OR Lung OR Neoplasm* OR Tumor* OR Malignancy OR Carcinoma)

3,329,786 documents found

TITLE ("Stem Cell*" OR "Cancer Stem Cell*" OR CSC OR "Tumor-initiating cell*" OR "Neoplastic stem cell*" OR "Anticancer cell*")

221,703 documents found

TITLE ("Therapy resistance" OR "Drug resistance" OR "Treatment resistance" OR Resistance OR Inhibit* OR Block* OR Mediate* OR "Car-T" OR "Immunotherap*" OR Therap* OR "Natural Killer Cell*")

3,605,130 documents found

c) Web of Science

#5	4,785	#1 AND #2 AND #3 and 2016 or 2015 or 2017 or 2018 or 2019 or 2020 or 2021 or 2022 or 2023 or 2024 or 2025 (Publication Years)			
# 4	7,028	#1 AND #2 AND #3<br>*Indexes=SCI-EXPANDED, SSCI, A&HCI, CPCI-S, CPCI-SSH, ESCI Timespan=All years*	Edit	☐	☐
# 3	3,518,148	TI=(“Therapy resistance” OR “Drug resistance” OR “Treatment resistance” OR Resistance OR Inhibit* OR Block* OR Mediate* OR “Car-T” OR "Immunotherap*" OR Therap* OR “Natural Killer Cell*”)	Edit	☐	☐
# 2	273,227	TI=(“Stem Cell*” OR “Cancer Stem Cell*” OR CSC OR “Tumor-initiating cell*” OR “Neoplastic stem cell*” OR “Anticancer cell*”)	Edit	☐	☐
# 1	3,351,143	TI=(Cancer* OR Breast OR Colorectal OR Lung OR Neoplasm* OR Tumor* OR Malignancy OR Carcinoma)	Edit	☐	☐

d) Cochrane database for clinical trial

cancer* OR breast OR colorectal OR lung OR neoplasm* OR tumor* OR malignancy OR carcinoma

189 797

(stem NEXT cell*) OR (cancer NEXT stem NEXT cell*) OR CSC OR (tumor-initiating NEXT cell*) OR (neoplastic NEXT stem NEXT cell*) OR (anticancer NEXT cell*)

7843

(therapy NEXT resistance) OR (drug NEXT resistance) OR (treatment NEXT resistance) OR resistance OR inhibit* OR block* OR mediate* OR CAR-T OR immunotherap* OR therap* OR (natural NEXT killer NEXT cell*)

264338

cancer* OR breast OR colorectal OR lung OR neoplasm* OR tumor* OR malignancy OR carcinoma in Record Title AND (stem NEXT cell*) OR (cancer NEXT stem NEXT cell*) OR CSC OR (tumor-initiating NEXT cell*) OR (neoplastic NEXT stem NEXT cell*) OR (anticancer NEXT cell*) in Record Title AND (therapy NEXT resistance) OR (drug NEXT resistance) OR (treatment NEXT resistance) OR resistance OR inhibit* OR block* OR mediate* OR CAR-T OR immunotherap* OR therap* OR (natural NEXT killer NEXT cell*) in Record Title - (Word variations have been searched)
Overall: 83

From 2015 to 2025: 29
