# Supplementary material for: Resistance of Colorectal Cancer Stem Cells to Modern Therapies: A Systematic Review
Source: Int J Mol Sci. 2026 Jul 15;27(14):6285. doi: 10.3390/ijms27146285 (PMC13409747; doi:10.3390/ijms27146285)
Supplement: Supplementary file 1 [file ijms-27-06285-s001.zip › Tables S1-S4.pdf]

Tables S1-S4. Risk of Bias Assessment.

Table S1. Quality assessment in accordance with the QUIN which was done by A.B.

Ref	First Author	AO	SSC	ST	CG	M	OD	R	OM	OAD	B	SA	PR	Total	Score (%)	RoB category
[18]	Wu et al., 2018	2	0	1	2	2	0	0	2	0	0	2	2	**13/24**	**54.2**	Medium
[19]	Geng et al., 2024	2	0	0	2	2	0	0	2	0	0	2	2	**12/24**	**50.0**	Medium
[20]	Qin et al., 2023	2	0	1	2	2	0	0	2	0	0	2	2	**13/24**	**54.2**	Medium
[21]	Guo et al., 2020	2	0	0	2	2	0	0	2	0	0	2	2	**12/24**	**50.0**	Medium
[22]	Roy et al., 2022	2	0	1	2	2	0	2	2	0	0	2	2	**15/24**	**62.5**	Medium
[23]	Chen et al., 2022	2	0	1	2	2	0	2	2	0	0	2	2	**15/24**	**62.5**	Medium
[24]	Chen et al., 2017	2	0	1	2	2	0	2	2	0	0	2	2	**15/24**	**62.5**	Medium
[25]	Wang et al., 2024	2	0	2	2	2	0	0	2	0	0	2	2	**14/24**	**58.3**	Medium
[26]	Huang et al., 2018	2	0	1	2	2	0	2	2	0	0	2	2	**15/24**	**62.5**	Medium
[27]	Lamichhane et al., 2022	2	0	1	2	2	0	0	2	0	0	2	2	**13/24**	**54.2**	Medium
[28]	Su et al., 2023	2	0	1	2	2	0	1	2	0	0	2	2	**14/24**	**58.3**	Medium
[29]	Kim et al., 2025	2	0	1	2	2	0	0	2	0	0	2	2	**13/24**	**54.2**	Medium
[30]	De Angelis et al., 2016	2	2	0	2	2	0	0	2	0	0	2	2	**14/24**	**58.3**	Medium
[31]	Di Franco et al., 2021	2	0	1	2	2	0	0	2	0	0	2	2	**13/24**	**54.2**	Medium
[32]	Gaggianesi et al., 2022	2	0	1	2	2	0	0	2	0	0	2	2	**13/24**	**54.2**	Medium
[33]	Prasetyanti et al., 2015	2	0	1	2	2	0	1	2	0	0	2	2	**14/24**	**58.3**	Medium
[34]	Rio-Vilariño et al., 2024	2	0	1	2	2	0	2	2	0	0	2	2	**15/24**	**62.5**	Medium
[35]	Lepore Signorile et al., 2025	2	0	1	2	2	0	1	2	0	0	2	2	**14/24**	**58.3**	Medium

[36]	Mattiello et al., 2021	2	0	1	2	2	0	0	2	0	0	2	2	**14/24**	**58.3**	Medium
[37]	Honma et al., 2019	2	0	1	2	2	0	1	2	0	0	2	2	**14/24**	**58.3**	Medium
[38]	Ghosh et al., 2025	2	0	1	2	1	0	0	2	0	0	2	2	**12/24**	**50.0**	Medium
[39]	Paul et al., 2023	2	0	2	2	2	0	0	2	0	0	2	2	**14/24**	**58.3**	Medium
[40]	Huynh et al., 2016	2	0	1	2	2	0	1	2	0	0	2	2	**14/24**	**58.3**	Medium
[41]	Planque et al., 2016	2	0	1	2	2	0	2	2	0	0	2	2	**15/24**	**62.5**	Medium
[42]	Bergin et al., 2024	2	0	1	2	2	0	1	2	0	0	2	2	**14/24**	**58.3**	Medium
[43]	Huang et al., 2020	2	0	1	2	2	0	2	2	0	0	2	2	**15/24**	**62.5**	Medium

Note: AO, aims/objectives; SSC, sample size calculation; ST, sampling technique; CG, comparison group; M, methodology; OD, operator details; R, randomisation; OM, outcome measurement; OAD, outcome assessor details; B, blinding; SA, statistical analysis; PR, presentation of results; RoB, risk of bias. QUIN criteria were scored as follows: 2 = adequately specified, 1 = inadequately specified, 0 = not specified, and NA = not applicable. Criteria marked as NA were excluded from the denominator. The final score was calculated using the formula: Final score = (total score × 100) / (2 × number of applicable criteria). Studies were categorised as low risk of bias (>70%), medium risk of bias (50–70%), or high risk of bias (<50%).

Table S2. Quality assessment in accordance with the QUIN which was done by S.R.

Ref	First Author	AO	SSC	ST	CG	M	OD	R	OM	OAD	B	SA	PR	Total	Score (%)	RoB category
[18]	Wu et al., 2018	2	0	1	2	2	0	0	2	0	0	2	2	**13/24**	**54.2**	Medium
[37]	Honma et al., 2019	2	0	1	2	2	0	0	2	0	0	2	2	**13/24**	**54.2**	Medium
[34]	Rio-Vilariño et al., 2024	2	0	1	2	2	0	1	2	0	0	2	2	**14/24**	**58.3**	Medium
[32]	Gaggianesi et al., 2022	2	0	1	2	2	0	0	2	0	0	2	2	**13/24**	**54.2**	Medium
[41]	Planque et al., 2016	2	0	1	2	2	0	2	2	0	0	2	2	**15/24**	**62.5**	Medium

Note: AO, aims/objectives; SSC, sample size calculation; ST, sampling technique; CG, comparison group; M, methodology; OD, operator details; R, randomisation; OM, outcome measurement; OAD, outcome assessor details; B, blinding; SA, statistical analysis; PR, presentation of results; RoB, risk of bias. QUIN criteria were scored as follows: 2 = adequately specified, 1 = inadequately specified, 0 = not specified, and NA = not applicable. Criteria marked as NA were excluded from the denominator. The final score was calculated using the formula: Final score = (total score × 100) / (2 × number of applicable criteria). Studies were categorised as low risk of bias (>70%), medium risk of bias (50–70%), or high risk of bias (<50%).

Table S3. Quality assessment in accordance with the SYRCLE which was done by A.B.

Ref	Author	SG	BC	AC	RH	BI	ROA	BOA	IOD	SOR	OB
[28]	Su et al., 2023	No	Unclear	Unclear	Unclear	Unclear	Unclear	Unclear	Yes	Yes	No
[30]	De Angelis et al., 2016	Unclear	No	Unclear	Unclear	Unclear	Unclear	Unclear	Yes	Yes	Unclear
[31]	Di Franco et al., 2021	Unclear	No	Unclear	Unclear	Unclear	Unclear	Unclear	Yes	Yes	No
[20]	Qin et al., 2023	Unclear	No	Unclear	Unclear	Unclear	Unclear	Unclear	Yes	Yes	No
[32]	Gaggianesi et al., 2022	No	Yes	Unclear	No	Unclear	No	Unclear	Yes	Yes	No
[21]	Guo et al., 2020	No	Yes	Unclear	No	Unclear	No	Unclear	Yes	Yes	No
[33]	Prasetyanti et al., 2015	No	Yes	Unclear	No	Unclear	No	Unclear	Yes	Yes	No
[37]	Honma et al., 2019	No	Yes	Unclear	No	Unclear	No	Unclear	Yes	Yes	No
[26]	Huang et al., 2018	No	Yes	Unclear	No	Unclear	No	Unclear	Yes	Yes	No
[34]	Rio-Vilariño et al., 2024	No	Yes	Unclear	No	Unclear	No	Unclear	Yes	Yes	No
[38]	Ghosh et al., 2025	No	Yes	Unclear	No	Unclear	No	Unclear	Yes	Yes	No
[43]	Huang et al., 2020	No	Yes	Unclear	No	Unclear	No	Unclear	Yes	Yes	No
[41]	Planque et al., 2016	No	Yes	Unclear	No	Unclear	No	Unclear	Yes	Yes	No

[35]	Lepore Signorile et al., 2025	No	Yes	Unclear	No	Unclear	No	Unclear	Yes	Yes	No
[42]	Bergin et al., 2024	No	Yes	Unclear	No	Unclear	No	Unclear	Yes	Yes	No
[40]	Huynh et al., 2016	No	Yes	Unclear	No	Unclear	No	Unclear	Yes	Yes	No
[24]	Chen et al., 2017	Unclear	Yes	Unclear	No	Unclear	No	Unclear	Yes	Yes	No
[39]	Paul et al., 2023	No	No	No	Yes	Unclear	No	Unclear	Yes	Yes	No
[25]	Wang et al., 2024	No	No	Unclear	No	Unclear	No	Unclear	Yes	Yes	No
[23]	Chen et al., 2022	No	No	Unclear	No	Unclear	No	Unclear	Yes	Yes	No

Note: SG, sequence generation; BC, baseline characteristics; AC, allocation concealment; RH, random housing; BI, blinding of caregivers/investigators; ROA, random outcome assessment; BOA, blinding of outcome assessor; IOD, incomplete outcome data; SOR, selective outcome reporting; OB, other bias. SYRCLE judgements are presented as Yes/No/Unclear according to the tool's signalling-question format. Yes indicates low risk of bias, No indicates high risk of bias, and Unclear indicates insufficient reporting to permit a judgement. No summary score was calculated, in accordance with SYRCLE guidance.

Table S4. Quality assessment in accordance with the SYRCLE which was done by S.R.

Ref	Author	SG	BC	AC	RH	BI	ROA	BOA	IOD	SOR	OB
[32]	Gaggianesi et al., 2022	Unclear	Yes	Unclear	Unclear	Unclear	Unclear	Unclear	Yes	Unclear	Yes
[30]	De Angelis et al., 2016	Unclear	Yes	Unclear	Unclear	Unclear	Unclear	Unclear	Yes	Unclear	Yes
[38]	Ghosh et al., 2025	Unclear	Yes	Unclear	Unclear	Unclear	Unclear	Unclear	Yes	Unclear	Yes
[42]	Bergin et al., 2024	Yes	Yes	Unclear	Unclear	Yes	Unclear	Yes	Yes	Unclear	Yes

Note: SG, sequence generation; BC, baseline characteristics; AC, allocation concealment; RH, random housing; BI, blinding of caregivers/investigators; ROA, random outcome assessment; BOA, blinding of outcome assessor; IOD, incomplete outcome data; SOR, selective outcome reporting; OB, other bias. SYRCLE judgements are presented as Yes/No/Unclear according to the tool's signalling-question format. Yes indicates low risk of bias, No indicates high risk of bias, and Unclear indicates insufficient reporting to permit a judgement. No summary score was calculated, in accordance with SYRCLE guidance.
